# Supplementary material for: Speckle-illumination spatial frequency domain imaging with a stereo laparoscope for profile-corrected optical property mapping
Source: J Biomed Opt. 2025 Jan 24;30(Suppl 1):S13710. doi: 10.1117/1.JBO.30.S1.S13710 (PMC11759297; doi:10.1117/1.JBO.30.S1.S13710)
Supplement: Supplementary file 1 [file JBO_030_S13710_SD001.pdf]

Supplementary information

Laser speckle illumination depth of field (DOF)

Figure S1 shows the laser speckle contrast ($K = \sigma/\mu$) as a function of distance from the laparoscope imaging tip. Measurements were taken from 2cm to 20cm on a piece of white printer paper. We extracted a 250x250-pixel patch from the center of the laparoscope camera sensor. This patch was flat-field corrected, then Gaussian blurred with a kernel size of 3 pixels to measure the speckle contrast K. We observed that K was within 85% of the max value over a 4cm to 10cm working distance range. Below 4cm and above 10cm, there is a decrease in the speckle contrast due to blurring of the speckle pattern from the limited DOF of the imaging optics in the laparoscope. The illumination divergence approximately matches the imaging angular field of view, such that the imaged speckle size is constant with working distance, while the object speckle size scales with Equation 1.

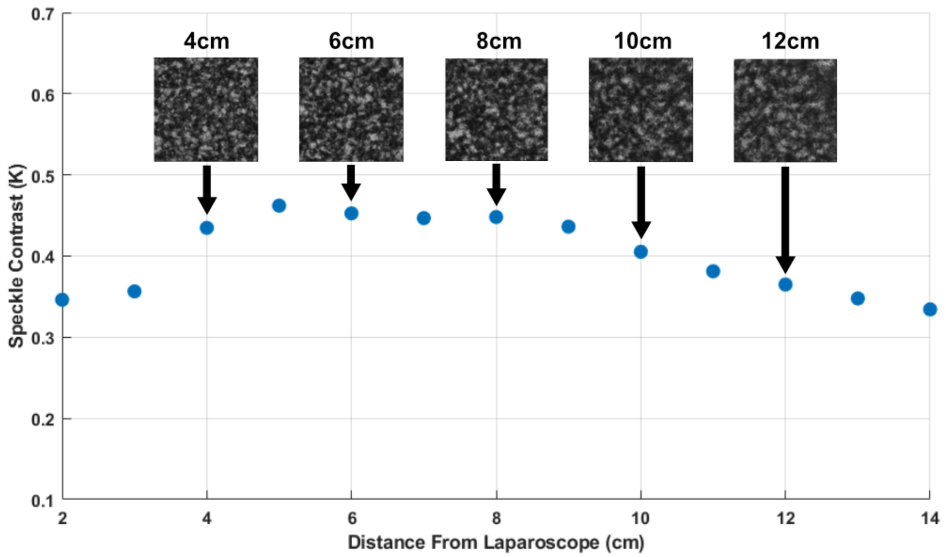

Figure S1. Laser speckle contrast (K) as a function of distance from the laparoscope tip. The K is within 90% of the peak value from 4 to 10cm.
